# Pathogen-specific T Cells: Targeting Old Enemies and New Invaders in Transplantation and Beyond

**DOI:** 10.1097/HS9.0000000000000809

**Published:** 2023-01-09

**Authors:** Anastasia Papadopoulou, Maria Alvanou, George Karavalakis, Ifigeneia Tzannou, Evangelia Yannaki

**Affiliations:** 1 Hematology Department, Hematopoietic Cell Transplantation Unit, Gene and Cell Therapy Center, “George Papanikolaou” Hospital, Thessaloniki, Greece; 2 University General Hospital of Patras, Greece; 3 Department of Hematology and Lymphomas and Bone Marrow Transplantation Unit, Evangelismos General Hospital, Athens, Greece

## Abstract

Adoptive immunotherapy with virus-specific cytotoxic T cells (VSTs) has evolved over the last three decades as a strategy to rapidly restore virus-specific immunity to prevent or treat viral diseases after solid organ or allogeneic hematopoietic cell-transplantation (allo-HCT). Since the early proof-of-principle studies demonstrating that seropositive donor-derived T cells, specific for the commonest pathogens post transplantation, namely cytomegalovirus or Epstein-Barr virus (EBV) and generated by time- and labor-intensive protocols, could effectively control viral infections, major breakthroughs have then streamlined the manufacturing process of pathogen-specific T cells (pSTs), broadened the breadth of target recognition to even include novel emerging pathogens and enabled off-the-shelf administration or pathogen-naive donor pST production. We herein review the journey of evolution of adoptive immunotherapy with nonengineered, natural pSTs against infections and virus-associated malignancies in the transplant setting and briefly touch upon recent achievements using pSTs outside this context.

## INTRODUCTION

Opportunistic infections/reactivations remain the Achilles heel of allogeneic hematopoietic cell transplantation (allo-HCT) significantly increasing morbidity and mortality among allograft recipients.^1–4^ Despite routine molecular viral monitoring and the extensive application of antiviral prophylaxis and preemptive therapy with pharmacological agents, common viruses such as cytomegalovirus (CMV), Epstein-Barr virus (EBV), adenovirus (AdV), BK virus, as well as fungi, mainly *Aspergillus fumigatus* (AF) are major clinical concerns. Prolonged immune deficiency and especially delayed T-cell reconstitution posttransplant plays an important role in the outcome of antiviral prophylaxis and treatment, affecting the transplant-related mortality, all-cause mortality, and even relapse.^3,4^ To address this major limitation, sophisticated antiviral approaches have been explored, including adoptive transfer of T cells. In the current review, we describe the preclinical and clinical achievements of adoptive immunotherapy with pathogen-specific T cells (pSTs) in the allo-HCT setting and discuss the challenges and striking developments toward broader application. We also summarize recent achievements using nonengineered and genetically engineered pSTs against infections and virus-associated malignancies, beyond the transplant setting.

## SINGLE-TARGETING PSTS AGAINST THE MOST COMMON PATHOGENS IN THE TRANSPLANT SETTING

In 1994, 5 patients with monoclonal EBV-post-transplant lymphoproliferative disease (EBV-PTLD) achieved complete remission after the infusion of peripheral blood mononuclear cells (PBMCs) from their EBV-seropositive transplant donors, demonstrating that donor lymphocyte infusions (DLIs) are effective in restoring virus-specific immunity and controlling viral infection.^5^ Nevertheless, broader application of DLIs in this context has been limited mainly due to their low content in virus-specific T cells compared with the higher frequency of alloreactive T cells that can cause severe graft-versus-host-disease (GvHD).^6^ To circumvent such an unwanted event, the use of conditionally eliminated DLIs being genetically modified to express suicide genes has been explored providing promising results.^7^

Later on, Rooney et al prepared and administered the first donor-derived, EBV-specific cytotoxic T-lymphocyte lines (EBV-CTLs), successfully preventing or controlling EBV reactivation in ten allograft recipients, without evidence of GvHD.^8^ In a multicenter study, Heslop et al. demonstrated high efficacy of EBV-CTLs as prophylaxis (0/101 patients developed PTLD) or treatment of PTLD (11/13 patients controlled PTLD) in allograft recipients and also persistence of functional CTLs for up to 9 years.^9^

**Figure 1.**
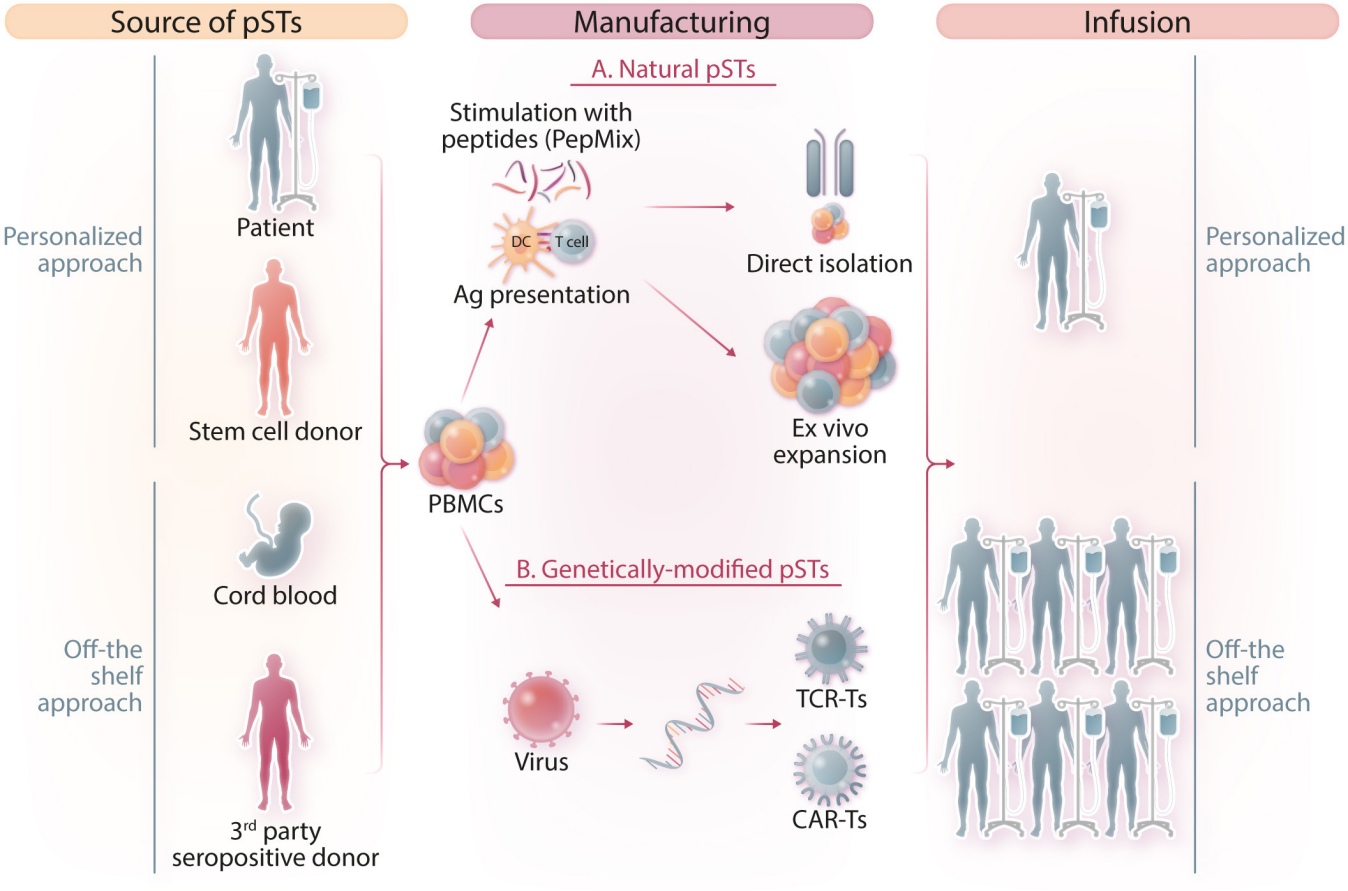
**Strategies for adoptive immunotherapy with pSTs.** The starting material for the generation of pSTs is the mononuclear cell population isolated from different donor sources. In personalized adoptive immunotherapy, either the patient or the HLA-matched stem cell donor serves as the cell source (autologous or allogeneic, respectively). In off-the-shelf adoptive immunotherapy, pSTs are derived either from cord blood or selected sepositive donor banked blood. T cells can then be stimulated via viral peptide/protein/lysate or antigen-loaded antigen-presenting cells and pSTs are either directly isolated or ex vivo expanded, as natural pSTs (A) or T cells are transduced with a viral vector to express either a transgenic TCR of specific targeting or a CAR redirecting the specificity of T cells against a certain target pathogen. (B) Each either natural or genetically engineered pST cell product can be administered in the transplantation setting to a single, HLA-matched patient (personalized therapy, one donor-one recipient) or multiple, partially HLA-matched patients, within or outside the transplantation context (off-the-shelf therapy, one donor-multiple recipients). CAR = chimeric antigen receptor; pSTs = pathogen-specific T cells; TCR = T-cell receptor.

Despite efficacy, the widespread use of EBV-CTLs was severely limited by the 8–12 weeks manufacturing time and the process complexity,^10^ major obstacles for timely delivery to a patient developing PTLD. Since then, several groups have explored the development of faster and less sophisticated approaches and the manufacturing process has been significantly shortened and simplified. Such approaches involve (i) direct selection of donor VSTs with multimers that select T cells directed against specific viral peptides in the context of a specific HLA class I molecule^11^ *or* IFN-γ capture, where specific T cells are selected on the basis of IFN-γ secretion upon viral stimulation^12^ and (ii) ex vivo expansion of the endogenous antigen-specific T cells by specific T-cell stimulation with mixtures of overlapping peptides^13^ (Figure 1).

CMV-specific T-cell generation went also through various stages of protocol refinements. In the first proof-of-principle study by Riddell et al, virus-specific T-cell clones from a healthy donor generated ex vivo from autologous CMV-infected fibroblasts and adoptively transferred into an allo-HCT recipient, prevented CMV infection without causing GvHD.^14^ In another early study, Walter et al used fibroblasts infected with the AD169 strain of CMV to stimulate donor T cells followed by limiting-dilution cloning to isolate cytolytic CD8+ CMV-directed T cells for adoptive transfer. The CMV-specific clones were administered prophylactically to 14 recipients of HLA-matched sibling donor grafts in weekly escalated doses. All patients developed early on, CMV-specific T-cell reconstitution showing in their majority (11/14) significantly increased cytotoxic activity against CMV in vitro. GvHD developed in 7 patients before the first infusion, in 3 patients during the T-cell therapy course or after the last dose, while 4 patients did not develop GvHD. Neither CMV viremia nor disease developed in any treated patient.^15^

Also, Perruccio et al generated CD4^+^CMV-specific T-cell clones using donor PBMCs stimulated with CMV lysate, which were infused in 25 haploidentical HCT recipients. Cellular therapy proved to be safe and efficient in accelerating the recovery of endogenous CD8+CMV-specific responses, clinically translating into fewer CMV reactivation episodes and lower antigenemia levels.^16^

Alternatively, Einsele et al generated both CD4^+^- CD8^+^-CMV-specific CTLs using donor PBMCs pulsed with CMV lysate. The cellular products were safely infused to 8 HCT recipients who presented ganciclovir-resistant CMV viremia. Significant viral load reduction was observed in 7 patients, whereas in the 2 patients with the highest viral loads, the antiviral effect was only transient. A second T-cell infusion in one of the 2 patients resulted in complete response.^17^ Similar results with excellent response rates were reported by other groups as well.^12,18^

Many studies targeted AdV, another common pathogen, especially in the pediatric HCT field. Feuchtinger et al isolated AdV-specific donor T cells based on IFN-γ secretion after in vitro antigenic stimulation. The cellular products which combined both CD4^+^ and CD8^+^ T cells were infused into 9 children with systemic AdV infection post-HCT. Five of 6 evaluable patients managed to successfully clear viremia. T-cell infusion was well tolerated in all patients, except one case with skin GvHD grade II.^19^ In another phase I/II multicenter study, Ip et al demonstrated safety and feasibility of the preemptive administration of AdV-specific T cells in high-risk pediatric patients after allo-HCT to control viremia. Eight children received the cellular products and all successfully controlled the infection, while 1 patient developed stage II GvHD 2 months postinfusion.^20^ Feucht et al showed that adoptive transfer of TH1 cells is a safe and effective alternative therapeutic approach for refractory AdV disease or viremia after HCT, resulting, in patients with antigen-specific T-cell responses (14/23), in 86% (12/14) complete remissions and 100% survival (versus 9.5% in nonresponders).^21^ Clinical trials and preclinical studies with pSTs are summarized in Tables 1, 2.

**Table 1:** Clinical Trials With Single- and Multitargeting Pathogen-specific T Cells

	Targeted Pathogen(s)	Manufacturing	Pts	Prophylaxis/Treatment	Dose	Safety	Clinical Response	References
Donor-derived	CMV	Ex vivo expansion	14	Prophylaxis	33 × 10^6^–1 × 10^9^/m^2^	3/14 GvHD grade I-II	14/14 CR	Walter et al^15^
	CMV	Ex vivo expansion	8	Treatment	1 × 10^7^/m^2^	No toxicity	6/8 CR, 1/8 PR, 1/8 NR	Einsele et al^17^
	CMV	Ex vivo expansion	30	Prophylaxis	0.6–1 × 10^5^/kg	7/30 aGvHD grade I, 4/30 aGvHD grade II-III, 12/28 cGvHD	27/27 CR, 20/27 required antivirals	Peggs et al^18^
	CMV	Ex vivo expansion	50	Prophylaxis	2 × 10^7^/m^2^	7/50 aGvHD, 1 CMV-related death	26/50 reactivations, 5/26 reactivations post infusion, 1/5 required antivirals	Blyth et al^22^
	CMV	Ex vivo expansion	32	Treatment	CD8+: 0.66–15.41 × 10^7^, CD4+: 0.68–9.25 × 10^5^	1/32 GvHD grade II	27/32 CR, 5/32 NR	Pei et al^23^
	CMV	Ex vivo expansion and DC vaccine	4	Prophylaxis	2 × 10^7^/m^2^, DC 19 × 10^6^	1/4 aGvHD grade III, 3/4 cGvHD	3/4 CR	Ma et al^24^
	CMV	IFN-gamma capture	18	Prophylaxis/ preemptive	1 × 10^4^/kg	7/18 aGVHD grade I-II, 1/18 aGVHD grade III, 3/18 cGvHD	1/7 pts treated prophylactically reactivated - 11/11 pts treated preemptively cleared CMV	Peggs et al^12^
	CMV	Streptamer selection	2	Treatment	0.37–2.2 × 10^5^/kg	No toxicity	2/2 CR	Schmitt et al^25^
	CMV	Streptamer selection	2	Treatment	3.7–5.1 × 10^3^/kg	No toxicity	2/2 CR	Stemberger et al^26^
	CMV	Tetramer selection	9	Treatment	1.2 × 10^3^–3.3 × 10^4^/kg	No toxicity	8/9 CR, 1/9 PR	Cobbold et al^27^
	CMV	Ex vivo expansion	9	Prophylaxis	2 × 10^7^/m^2^	3/9 aGvHD grade I-II, 2/9 cGvHD	7/9 CR, 2/9 CMV reactivations	Mickleithwaite et al^28^
	CMV	Ex vivo expansion	12	Prophylaxis	2 × 10^7^/m^2^	4/12 GvHD	4/12 CMV reactivations	Micklethwaite et al^29^
	CMV	Ex vivo expansion	16	Prophylaxis	0.5–1 × 10^5^	3/16 GvHD grade I	2/16 CMV reactivations	Peggs et al^30^
	CMV	Ex vivo expansion	7	Treatment	2.5–5 × 10^5^/kg	No toxicity	4/7 CR, 2/7 PR, 1/7 NR	Bao et al^31^
	CMV	IFN-gamma capture	6	Treatment	0.9 × 10^4^–3.1 × 10^5^/kg	No toxicity	6/6 CR	Meij et al^32^
	CMV or EBV	Ex vivo expansion	3	Treatment	10^6^ cells/kg (3–8 infusions)	1/3 aGvHD grade II	CMV: 3/3 CR; EBV: 1/1 CR	Dong et al^33^
	CMV or AdV	IFN-gamma capture	15	Treatment	3.5–3.7 × 10^3^/kg	1/15 GvHD grade III, 2/15 septic shock, 4/15 worsening of respiratory symptoms or liver cytolysis	CMV 3/8 CR, 5/8 NR; AdV 3/5 CR, 1/5 PR, 1/5 NR	Creidy et al^34^
	EBV	Ex vivo expansion	114	Prophylaxis/ treatment	2–5 × 10^7^/m^2^	8/114 aGvHD, 13/114 cGvHD	Prophylaxis: 101/101 CR, treatment: 11/13 CR, 2/NR	Rooney, Heslop^8,9,35–37^
	EBV	Ex vivo expansion	6	Treatment	1 × 10^7^/m^2^ (2–4 infusions)	1/6 aGvhD limited reactivation	5/6 PR, 1/6 NR	Gustafsson et al^38^
	EBV	Ex vivo expansion	4	Treatment	35 × 10^6^/m^2^	No toxicity	3/4 CR	Comoli et al^39^
	EBV	Ex vivo expansion	6	Treatment	0.5 × 10^6^/kg	No toxicity	6/6 CR	Comoli et al^40^
	EBV	IFN-gamma capture	6	Treatment	0.4–9.7 × 10^4^/kg	No toxicity	3/6 CR, 3/6 NR	Moosman et al^41^
	EBV	IFN-gamma capture	10	Treatment	1.5 × 10^2^–5.4 × 10^4^/kg	1/10 aGvhD grade I-II	6/10 CR, 1/10 PR, 3/10 NR	Icheva et al^42^
	EBV	n/a	1	Treatment	8.2 × 10^6^–1 × 10^7^	No toxicity	1/1 NR	Imashuku et al^43^
	AdV	Ex vivo expansion	8	Preemptive	1 × 10^4^/kg	1/8 aGvHD	8/8 CR	Ip et al^20^
	AdV	IFN-gamma capture	9	Treatment	1.2 × 10^3^–5 × 10^4^/kg	1/6 aGvHD reactivation	3/6 CR, 2/6 PR, 1/6 NR	Feuchtinger et al^19^
	AdV	IFN-gamma capture	30	Treatment	0.3–24 × 10e3 cells/kg	2/30 aGvHD	18/29 CR, 3/29 PR, 8/29 NR	Feucht et al^44^
	AdV	Ex vivo expansion	2	Treatment	10^4^/kg	1/2 GvHD	1/2 CR, 1/2 NR	Geyqerreger et al^45^
	AdV	IFN-gamma capture	1	Treatment	34 × 10^3^ CD3+/kg 73 × 10^3^ CD8+/kg	No toxicity	1/1 CR	Di Nardo et al^46^
	AF or CMV (HCT)	Ex vivo expansion	35	Prophylaxis (CMV) or treatment (AF)	10^5^–3 × 10^6^ cells/kg	1/35 aGvHD grade II	AF: 9/10 CR; CMV: 7/25 reactivations	Perrucio et al^16^
	BKV (HCT)	IFN-γ capture	1	Treatment	0.34 × 10^4^ cells/kg	No toxicity	1/1 CR	Pello et al^47^
	JC (PML post-HCT)	Ex vivo expansion	1	Treatment	0.5 × 10^6^ and 10^6^ cells/kg	No toxicity	1/1 PR	Balduzzi et al^48^
	JC (PML post-HCT)	IFN-γ capture	1	Treatment	2 × 10^4^ total	No toxicity	1/1 CR	Steinhardt et al^49^
	EBV, AdV	Ex vivo expansion	13	Prophylaxis/ treatment	0.5–13.5 × 10^7^ cells/m^2^	No toxicity	EBV: 13/13 prophylaxis; AdV: 2/2 CR	Leen et al^50^
	CMV, EBV, AdV	Ex vivo expansion	11	Prophylaxis/ treatment	0.5–10 × 10^7^ cells/m^2^	No toxicity	CMV: 3/3; EBV: 3/3; AdV: 6/6	Leen et al^51^
	CMV, EBV, AdV	Ex vivo expansion	14	Prophylaxis/ treatment	0.5–2.5 × 10^7^ cells/m^2^	1/14 GvHD reactivation	CMV: 4/4; EBV: 1/2; Adv: 1/1	Abraham et al^52^
	CMV, EBV, AdV	Ex vivo expansion	10	Treatment	0.5–2 × 10^7^ cells/m^2^	1/10 possible GvHD grade II (skin)	CMV: 4/5 CR; EBV: ¾ CR; AdV: 5/5 CR	Gerdeman et al^53^
	CMV, EBV, AdV, VZV	Ex vivo expansion	10	Prophylaxis	2 × 10^7^ cells/m^2^	No toxicity	CMV: 2/2; EBV, AdV, VZV: no reactivation	Ma et al^54^
	CMV, EBV, AdV, BKV	Ex vivo expansion	38 HCT; 3 SOT	Treatment	5 × 10^7^ VSTs/m^2^ (up to 10 infusions)	No toxicity	HCT donor-derived: 17/19 CR; HCT-3rd party-derived: 17/18 CR; SOT-3rd party-derived: EBV: 2/2 CR; BK: 3/3 CR	Nelson et al^55^
	CMV, EBV, AdV, BKV	Ex vivo expansion	23	Prophylaxis	2 × 10^7^ cells/m^2^	2/23 GvHD grade II-III	11/23 (CMV: 3/4; EBV: 6/7; BK: 5/5); 7/23 no viral load	Rubinsten et al^56^
	CMV, EBV, AdV, BKV, HHV6	Ex vivo expansion	11	Prophylaxis/treatment	0.5–2 × 10^7^ cells/m^2^	1/11 GvHD grade II	CMV: 3/3; EBV: 5/5; AdV: 1/1; BKV: 6/7; HHV6: 2/2	Papadopoulou et al^57^
	CMV, EBV, AdV, BKV, VZV, AF, Influenza	Ex vivo expansion	11	Prophylaxis	2 × 10^7^ cells/m^2^	2/11 aGvHD grade I-II, 4/11 aGvHD grade III-IV	CMV: mixed; EBV: mixed; BKV: 10/10 CR; Influenza: 2/2 CR; AdV-AF-VZV: 0 developed	Gottllieb et al^58^
Donor- & third party-derived	CMV	Streptamer selection	16	Treatment	6.34–14.6 × 10^6^	1/16 aGvHD; 1/16 cGvHD	9/16 CR, 3/16 PR, 4/16 NR	Neuenhahn et al^59^
	CMV	Ex vivo expansion	17	Treatment	0.5–2 × 10^6^/kg	No toxicity	15/17 CR, 2/17 NR	Koehne et al^60^
	CMV	IFN-gamma capture	18	Treatment	1.2 × 10^3^–1.7 × 10^4^/kg	1/18 upper GI Bleeding	9/18 CR, 6/18 PR, 3/18 NR	Feuchtinger et al^61^
	CMV or EBV or AdV	Pentamer selection	8	Treatment	0.8–24.6 × 10^4^/kg	N/a	EBV-1/1 CR, ADV-1/1 NR, CMV-4/6 CR, 1/6 PR, 1/6 NR	Uhlin et al^62^
	EBV	Ex vivo expansion	19	Treatment	0.5–3.5 × 10^6^/kg	No toxicity	13/19 CR, 6/19 NR	Doubrovina et al^63^
	AdV	IFN-gamma capture	5	Treatment	10^4^–10^5^/kg	No toxicity	3/5 CR, 2/5 NR	Qasim et al^64^
	AdV	IFN-gamma capture	11	Treatment	0.25–9.38 × 10^3^/kg	3/11 GvHD reactivation	9/11 CR, 2/11 NR	Gerdemann et al^53^
	JC (PML; 5 post-HCT)	Ex vivo expansion	9	Treatment	1–2 × 10^5^ cells/kg (up to 6 infusions)	1/9 died VZV infection	4/9 PR; 1/9 SD; 3/9 PD; 1/9 died VZV infection	Berzero et al^65^
Third party-derived	CMV	Ex vivo expansion	10	Treatment	2 × 10^7^/m^2^	No toxicity	7/10 CR, 3/10 PR	Tzannou et al^66^
	CMV	IFN-gamma capture	2	Treatment	0.8–4.4 × 10^4^/kg	No toxicity	1/2 CR, 1/2 PR	Alonso et al^67^
	EBV	Ex vivo expansion	33	Treatment	9–18 × 10^6^/kg	1/33 aGvHD grade I	19/33 CR, 3/33 PR, 1/33 SD, 9/33 PD	Prockop et al^68^
	EBV	Ex vivo expansion	11	Treatment	1–2 × 10^6^/kg	1/11 aGvHD	8/11 CR, 1/11 PR, 2/11 NR	Vickers et al^69^
	EBV	Ex vivo expansion	11	Treatment	5 × 10^6^/kg	No toxicity	3/11 CR, 1/11 PR, 7/11 NR	Gallot et al^70^
	EBV (1 HCT–7 SOT)	Ex vivo expansion	8	Treatment	10^6^ cells/kg (up to 6 infusions)	No toxicity	3/6 CR; 1/6 PR; 2/6 NR; 2 passed away before evaluation (unrelated to VSTs)	Haque et al^71^
	EBV (2 HCT–31 SOT)	Ex vivo expansion	33	Treatment	2 × 10^6^ cells/kg (up to 8 infusions)	No toxicity	12/33 CR; 9/33 PR; 12/33 NR	Haque et al^72^
	EBV (HCT)	Ex vivo expansion	2	Treatment	10^6^ cells/kg (5–6 infusions)	No toxicity	2/2 CR	Barker et al^73^
	BKV (renal transplant)	Ex vivo expansion	1	Treatment	3.6 × 10^7^ cells total (10 infusions)	No toxicity	1/1 CR	Jahan et al^74^
	BKV (HCT)	Ex vivo expansion	59	Treatment	1–2 × 10^5^ cells/kg (up to 5 infusions)	1/59 aGvHD Grade II (skin), 1/59 aGrade III GvHG (GI), 9/59 cGvHD	34/49 CR; 6/49 PR; 9/49 NR	Olson et al^75^
	BK (JC-PML) –not HCT	IFN-γ capture	2	Treatment	2.5 × 10^4^ cells/kg (3–4 infusions)	N/a	2/2 PR	Hopfner et al^76^
	BK (JC (PML) (1 HCT pt–2 not HCT)	Ex vivo expansion	3	Treatment	2 × 10^5^ cells/kg (up to 3 additional infusions)	No toxicity	2/3 PR, 1/3 SD	Muftuoglu et al^77^
	BK (JC (PML) –not HCT	Ex vivo expansion	12	Treatment	10^6^ cells/kg (plus up to 2 doses of 2 × 10^6^ cells/kg)	No toxicity	7/12 survived beyond 12 m	Cortese et al^78^
	SARS-CoV-2	Ex vivo expansion	57	Treatment	2 × 10^7^ cells/m^2^	No toxicity	Increased likelihood of recovery, lower risk of mortality than SoC alone	Papadopoulou et al^79^
	SARS-CoV-2	Memory T-cell selection	9	Treatment	0.1–1 × 10^6^ cells/kg	No toxicity	9/9 improvement	Perez-Martinez et al^80^
	SARS-CoV-2	Ex vivo expansion	1	Treatment	2 × 10^7^ cells (3 infusions)	No toxicity	1/1 CR	Martits-Chalangari et al^81^
	CMV, AdV or CMV, EBV or CMV or EBV or AdV	IFN-γ capture	9	Treatment	32–204.8 × 10^3^ cells/kg	1/9 cytokine storm	CMV: 4/5 CR; AdV: 3/3 CR; EBV: 3/3 CR	Kallay et al^82^
	CMV, EBV, AdV	Ex vivo expansion	50	Treatment	2 × 10^7^ cells/m^2^ (up to 6 infusions)	8/50 aGvHD (2 de novo)	CMV: 14/18 CR; EBV: 6/9 CR; AdV: 17/23 CR	Leen et al^83^
	CMV, EBV, AdV	Ex vivo expansion	30	Treatment	2–5 × 10^7^ cells/m^2^ (up to 3 infusions)	2/30 aGvHD grade II-IV; 5/30 cGvHD	CMV: 27/28 CR; EBV: 0/1 CR; AdV: 1/1 CR	Whithers et al^84^
	CMV, EBV, AdV, BKV	Ex vivo expansion	1	Treatment	5 × 10^7^ cells/m^2^ (2 infusions)	1/1 CRS	1/1 NR	Holland et al^85^
	CMV, EBV, AdV, BKV	Ex vivo expansion	4	Treatment PML (HCT setting)	5 × 10^7^ cells/m^2^ (up to 6 infusions)	No GvHD	1/4 SD; 3/4 PD	Rubinstein et al^86^
	CMV, EBV, AdV, BKV, HHV6	Ex vivo expansion	38	Treatment	2 × 10^7^ cells/m^2^ (up to 3 infusions)	6/38 aGvHD (3 de novo)	BKV: 20/20 CR; HHV6: 3/4 CR, 1/4 NE (not evaluated); CMV: 18/19 CR; EBV: 2/2 CR; ADV: 6/8 CR	Tzannou et al^87^
Autologous	MCPyV	Ex vivo expansion	1	Treatment	10^10^/m^2^ (3 infusions)	No toxicity	tumor regression in 2 of 3 detectable metastases	Chapuis et al^88^
	MCPyV	Ex vivo expansion	2	Treatment	10^10^/m^2^ (2–4 infusions)	Transient fevers lasting <24 h	2/2 CR, 2/2 late relapse	Paulson et al^89^
	HPV (no hct)	Ex vivo expansion from fragments of metastatic tumors (tumor-infiltrating T cells)	29	Treatment	9–152 × 10^9^ total	No toxicity	Cervidal cancer: 3/18 PR, 2/18 CR - noncervical cancer: 2/11 PR	Stevanović et al^90^
	HIV	Ex vivo expansion	1	Treatment	12–13 × 10^9^ (2 infusions)	N/a	1/1 PD	Koenig et al^91^
	HIV	Ex vivo expansion	6	Treatment	1 × 10^9^ total	No toxicity	3/6 PR	Lieberman et al^92^
	HIV	Ex vivo expansion	3	Treatment	3.3–33 × 10^8^ cells/m^2^ (5 infusions)	No toxicity	0/3 NR	Brodie et al^93^
	HIV	Ex vivo expansion	1	Treatment	1–1.7 × 10^9^ total (2 infusions)	No toxicity	1/1 SD	Tan et al^94^
	HIV	Ex vivo expansion	7	Treatment	3.3 × 10^9^ total	N/a	7/7 SD	Chapuis et al^95^
	HIV	Ex vivo expansion	6	Treatment	2 × 10^7^ cells/m^2^ (2 infusions)	No toxicity	6/6 SD (No detectable impact on the latent reservoir)	Sung et al^96^

AdV = adenovirus; AF = Aspergillus fumigatus; aGvHD = acute graft-versus-host-disease; BKV = BK virus; CMV = cytomegalovirus; CR = complete response; CRS = cytokine release syndrome; EBV = Epstein-Barr virus; GvHD = graft-versus-host-disease; HCT = hematopoietic cell transplantation; HHV6 = human herpesvirus 6; HIV = human immunodeficiency virus; HPV = human papillomavirus; IFN-*γ* = interferon-*γ*; MCPyV = Merkel cell polyomavirus; NR = no response; PBMCs = peripheral blood mononuclear cells; PD = progressive disease; PR = partial response; SARS-CoV-2 = severe acute respiratory syndrome coronavirus 2; SD = stable disease; VSTs = virus-specific T cells; VZV = varicella zoster.

**Table 2:** Preclinical Studies With Single- and Multitargeting Pathogen-specific T Cells

Targeted Pathogen(s)	Manufacturing	References
AF	Ex vivo expansion	Ramadan et al^97^
AF	IFN-γ capture	Beck et al^98^
AF	Ex vivo expansion	Zhu et al^99^
AF	IFN-γ capture and ex vivo expansion	Tramsen et al^100^
AF	Ex vivo expansion	Gaundar et al^101^
AF	CD137 selection and ex vivo expansion	Jolink et al^102^
AF	CD137 selection	Bacher et al^103^
AF	CD154 or CD137 selection and ex vivo expansion	Stuehler et al^104^
AF	Ex vivo expansion	Papadopoulou et al^123^
AF or *Aspergillus flavus* or *Aspergillus terreus* or *Candida albicans* or *Candida krusei* or *Fusarium solani* or *Fusarium oxysporum* or *Rhizopus oryzae* or *Lomentospora prolificans*	Ex vivo expansion	Deo et al^124^
BKV	Ex vivo expansion	Blyth et al^108^
BKV	Ex vivo expansion	Lamarche et al^110^
BKV	Ex vivo expansion	Wilhelm et al^109^
*C. albicans*	IFN-γ capture and ex vivo expansion	Tramsen et al^105^
HIV	Ex vivo expansion	Lam et al^128^
HIV	Ex vivo expansion	Patel et al^129^
HIV	Ex vivo expansion	Sung et al^96^
HIV	Ex vivo expansion	Patel et al^130^
HHV6	Ex vivo expansion	Gerdemann et al^112^
hMPV	Ex vivo expansion	Tzannou et al^116^
HPV	Ex vivo expansion	McCormack et al^120^
HPV	Ex vivo expansion	Van Poelgeest et al^119^
HPV	Ex vivo expansion	Ramos et al^118^
HPIV3	Ex vivo expansion	McLaughlin et al^115^
HPIV3	Ex vivo expansion	Harris et al^114^
HSV-1	Ex vivo expansion	Ma et al^117^
Influenza	Ex vivo expansion	Gaundar et al^113^
MCPyV	Ex vivo expansion	Davies et al^131^
Mycobacteria spp.	Ex vivo expansion	Patel et al^127^
Norovirus	Ex vivo expansion	Hanajiri et al^122^
*R. oryzae*	CD154 selection, ex vivo expansion, and IFN-γ enrichment	Schmidt et al^126^
*R. oryzae*	Ex vivo expansion	Castillo et al^125^
SARS-CoV-2	Ex vivo expansion	Keller et al^132^
SARS-CoV-2	Ex vivo expansion	Papayanni et al^133^
SARS-CoV-2	IFN-γ capture and ex vivo expansion	Cooper et al^134^
SARS-CoV-2	Ex vivo expansion	Kim et al^135^
SARS-CoV-2	Ex vivo expansion	Guerreiro et al^136^
SARS-CoV-2	Ex vivo expansion	Ferreras et al^80^
SARS-CoV-2	IFN-γ capture and ex vivo expansion	Garcia-Rios et al^137^
SARS-CoV-2	Ex vivo expansion	Pannikar et al^138^
VZV	Ex vivo expansion	Blyth et al^111^
Zika virus	Ex vivo expansion	Hanajiri et al^121^
CMV, EBV, AdV	Ex vivo expansion	Hanley et al^139^
CMV, EBV, AdV	Ex vivo expansion	Hanley et al^140^
CMV, EBV, AdV, BKV	Ex vivo expansion	Dasari et al^141^
CMV, EBV, AdV, BKV	Ex vivo expansion	Dave et al^142^
RSV, influenza, PIV, and hMPV	Ex vivo expansion	Vasileiou et al^143^
CMV, EBV, AdV, BKV, HHV6, VZV, JCV	Ex vivo expansion	Gerdeman et al^13^
AF, *C. albicans,* and *R. oryzae*	IFN-γ capture and ex vivo expansion post	Tramsen et al^144^
*A. terreus*, *C. krusei*, and *R. oryzae*	Ex vivo expansion ± TNF-α selection	Deo et al^124^
*C. krusei* and *A. terreus*	CD137 selection and ex vivo expansion	Castellano-Gonzalez et al^145^
AdV, EBV, CMV, *C. albicans*, and/or AF or AdV, EBV, *C. albicans*, and AF	CD154 selection and ex vivo expansion	Khanna et al^146^
EBV, CMV, BKV, and AF	Ex vivo expansion	Papadopoulou et al^147^

AdV = adenovirus; AF = Aspergillus fumigatus; BKV = BK virus; CMV = cytomegalovirus; EBV = Epstein-Barr virus; HHV6 = human herpesvirus 6; HIV = human immunodeficiency virus; hMPV = human metapneumovirus; HPV = human papillomavirus; HPIV3 = human parainfluenza virus-3; HSV-1 = herpes simplex virus type 1; IFN-*γ* = interferon-*γ*; MCPyV = Merkel cell polyomavirus; PIV = parainfluenza virus; RSV = respiratory syncytial virus; SARS-CoV-2 = severe acute respiratory syndrome coronavirus 2; VZV = varicella zoster.

## SINGLE-TARGETING PSTS AGAINST LESS COMMON PATHOGENS IN THE TRANSPLANT SETTING

The high response rates, along with the low toxicity observed in numerous early phase clinical trials of adoptively transferring VSTs (Table 1)

as prophylaxis or treatment against the main pathogens causing morbidity and mortality in immunocompromised hosts—namely CMV, EBV, and AdV—set the rationale for developing pSTs targeting less common, albeit well-recognized and potentially lethal for this particular population, opportunistic pathogens. In particular, since mid-2000s, manufacturing by ex vivo expansion or direct isolation, were separately employed or combined to enable the generation of AF- or *Candida albicans*-specific T-cell products.^97–105^ Nevertheless, clinical use of pSTs against mold infections was at that time hampered, due to the complex, lengthy, labor-intense, and costly manufacturing procedures.^106,107^

Major advances in synthetic overlapping peptide pools, use of cytokines and culture vessels/bioreactors, along with the in-depth characterization of immunogenic epitopes/antigens presented through human leukocyte antigen (HLA) molecules, have since then facilitated the production process and provided a springboard to extend the breadth of pathogens that can be targeted by T cells. Indeed, over the last years, the pST cell therapy platform has been simplified and adapted to an array of pathogens which complicate immune-deficient states, including viruses such as *BK polyomavirus (BKV*),^108–110^ h*uman herpesviruses* [*varicella zoster virus* (*VZV*),^111^ *human herpesvirus 6* (*HHV6*)^112]^, *respiratory viruses* [*influenza*,^113^ *human parainfluenza virus-3* (*HPIV3*),^114,115^ *human metapneumovirus* (*hMPV*)^116]^, *herpes simplex virus type 1* (*HSV-1*),^117^ *human papillomavirus* (*HPV*),^118–120^ *Zika virus*,^121^ *norovirus*,^122^ fungi such as *AF, Aspergillus flavus, Aspergillus terreus, C. albicans, Candida krusei, Fusarium solani, Fusarium oxysporum, Rhizopus oryzae, Lomentospora prolificans*^123–126^ and recently the more complex *Mycobacteria*^127^ (Table 2).

In the transplant setting, Perrucio et al^16^ reported that 9 of 10 haploidentical HCT patients treated with AF-specific T-cell clones survived invasive aspergillosis versus 7 of 13 individuals receiving conventional antifungal therapy. Other cases of transplanted patients treated with adoptive immunotherapy with donor-derived, monovalent virus-specific T cells (VSTs) against less common pathogens, included three patients who received in different studies, donor-derived JC-VSTs as treatment for the JC virus-associated progressive multifocal leukoencephalopathy (PML) and managed to clear JC achieving remarkable clinical improvement.^48,49,65^ In an another patient who was treated with IFN-γ capture-enriched BKV-STs from his HLA-haploidentical donor for drug-resistant BKV hemorrhagic cystitis, the treatment led to full resolution of symptoms and viremia.^47^ Those cases affirmed that the adoptive transfer of either isolated or ex vivo expanded T cells against less common pathogens, is safe and can restore pathogen-specific immune competence and control of opportunistic pathogen-associated diseases.

## MULTIVALENT PSTS AGAINST VARIOUS PATHOGENS IN THE TRANSPLANT SETTING

Despite the compelling results of single-targeting VSTs, transplanted patients usually experience reactivations from more than one opportunistic pathogens, thus making the generation of monovalent pSTs through multiple, time- labor-, and cost-intensive manufacturing processes, each time a new pathogen is being reactivated, rather impractical.

Leen et al pioneered the generation of bivalent and trivalent VSTs.^50,51^ By stimulating PBMCs with either a null adenoviral vector (Ad5f35^null^)^50^ or the Ad5f35pp65 vector encoding the CMV-pp65 antigen,^51^ and subsequently with autologous, EBV-transformed cell lines (EBV-LCLs) modified with the same vector(s) to artificially present adenoviral and/or (in the case of Ad5f35pp65) CMV-pp65 antigens, along with the constitutively expressed EBV antigens, demonstrated that the production of a single-cell product simultaneously targeting 2 or 3 viruses was feasible. Moreover, bi- or tri-VSTs presented clinically measurable antiviral activity after their administration to immunocompromised individuals, however, the long manufacturing procedure of 10 weeks limited wider applicability. By a similar approach, Hanley et al expanded tri-VSTs from virus-naive cord blood units (CBUs) using monocyte-derived dendritic cells (DCs) and Ad5f35pp65-transduced LCLs.^139^ The administration of tri-VSTs derived from a 20% fraction of the CBU to patients receiving a single CBU transplantation was safe and effective in preventing viral disease in the highest-risk patients.^52^ To simplify and accelerate the tri-VST production from seropositive donors, DCs were nucleofected with plasmids encoding CMV, EBV, and AdV antigens and used as antigen-presenting cells (APCs) for T-cell stimulation.^53^ This approach considerably shortened the manufacturing of tri-VSTs to 9–11 days.

Following the clinical success of tri-VSTs, expansion protocols for the production of tetravalent, pentavalent, and even heptavalent VSTs have been developed (Table 2), employing various APCs, including (i) DCs exposed to Ad5F35 encoding selected EBV epitopes and to VZV vaccine^54^; (ii) PBMCs infected with an AdV vector-based antigen presentation platform^141^; (iii) DCs pulsed with overlapping peptide pools of immunogenic viral proteins^13,57,143^; and (iv) phytohemagglutinin (PHA) blasts and K562 cells genetically modified to express co-stimulatory molecules.^142^ Among the various multi-VST generation methods, DCs-based approaches have been clinically tested, providing substantial responses as prophylaxis/treatment against VZV,^54^ EBV, CMV, AdV, BKV, and HHV6.^55–57^

Single- or multitargeting T-cell therapies against fungi have lagged well behind VSTs, mainly due to the complexity and length of the manufacturing procedures.^106,107^ Preclinical efforts, including ours, have been made towards the development of fungus-specific T-cell products,^124,144,145^ yet not clinically translated. To further unleash the potential of adoptive immunotherapy with antigen-specific T cells and enable the simultaneous treatment of multiple viral and fungal infections by an “all-in-one” single T-cell product, we and others, have developed refined strategies for manufacturing multipathogen-specific T-cell products (multi-pSTs). By either ex vivo expansion or magnetic isolation, T-cell products with specificity against a plethora of viruses and the main fungi affecting severely immunocompromised patients (AF and *Candida*)^148–151^ have been generated^146,147^ (Figure 1). In a clinical trial, multi-pSTs against 7 infectious pathogens, produced after combining and then expanding individually-primed cell products (AF-primed T cells combined with VZV-Influenza- and CMV-AdV-EBV-BKV-stimulated cultures) were administered prophylactically to allo-HCT recipients. This study provided the feasibility of multitargeting by a single-cell product; however, data interpretation could not be clear-cut, as the lack of a comparator group, the small sample size, and the prophylactic over therapeutic administration, did not allow firm conclusions about efficacy and safety. Indeed, it was impossible to distinguish whether GvHD occurrence in these patients was a de novo or multi-pST-induced event whereas the high-dose steroids needed to control GvHD in those patients, probably had rendered the infused multi-pSTs nonfunctional against the targeted-pathogens.^58^ We are currently evaluating in a clinical trial, the safety and efficacy of multi-pSTs simultaneously targeting AdV, CMV, EBV, BKV, and AF as treatment of corresponding infections post-allogeneic-HCT (EudraCT #2020-004725-23; NCT05471661).

There is accumulating evidence that AF and *C. albicans*, despite being profoundly different fungal pathogens, may induce CD4^+^ memory Th1 cells cross-protective against both pathogens, probably through molecular mimicry; the epitope p41 of the AF’s cell wall glucanase Crf1 has high homology to Chr1 epitope of Candida albicans, both being recognized by the same T-cell receptor (TCR).^152,153^ We and others have shown that such cross-reactivity may extend also to other *Aspergillus*, *Candida*, *Fusarium,* and *Mucorales* species,^101,104,123^ an important feature that will further augment the efficacy of multi-pSTs, induce a rapid restoration of a “broad repertoire” immunity, broaden the target patient population and reduce the manufacturing costs.

## CONFRONTING THE CHALLENGES TOWARD BROADER APPLICATION OF PSTS

### Donor unavailability and urgent need: the “third-party” approach

Multi-pSTs is an individualized product that for the time being can only be manufactured in specialized centers. Refined manufacturing procedures require at least 15 days for release, making these products unavailable for urgent administration, as in cases where an infection rapidly evolves or/and the stem cell donor is not accessible. The establishment of “off-the shelf,” third-party banks can help overcome such issues. With this strategy, pSTs are manufactured in advance from seropositive, transplant-eligible healthy donors, then being readily available for urgent use in partially HLA-matched patients with active infections (Figure 1).

The third-party bank approach was first studied in solid organ transplant (SOT) recipients where donor blood is not generally available for pST manufacture. Haque et al^72^ prepared EBV-STs from 100 healthy donors and treated 31 solid organ- and 2 HCT recipients with biopsy proven PTLD, refractory to conventional treatment. The patients received 1–6 infusions of 2 × 10^6^ VST cells/kg matched at 2–5 of 6 ΗLA loci with the patient. Despite the HLA disparity, infusions were well-tolerated without associated acute reactions, adverse effect on the transplanted organ or GvHD. Sixty-four percent of patients responded to treatment and 12 of 33 (36%) had a complete response. The investigators found an association between the number of matches and patient outcome. TCR spectratyping for in vivo tracking of the infused VSTs showed either a gradual increase in the peak signal with each infusion or a maximum signal, 4 or 24 hours after each infusion. The cells were detected until 7 days postinfusion. These results were recapitulated by several other groups.^68–70,73^ In a most recent study by the Memorial Sloan Kettering group, 33 HCT- and 13 SOT-individuals with rituximab-refractory PTLD, received 3 weekly VST infusions, with minimum 2 HLA allele-matching, followed by a 3-week observational period. The infusions were well-tolerated, but additional cycles were required for response (68% and 54% in the HCT and SOT recipients). In contrast to the study by Haque et al, this study showed no correlation between HLA matching and response.^68^

The use of multitargeting, off-the-shelf VSTs was first investigated by the Baylor group to treat patients with persistent CMV (n = 19), EBV (n = 18), and AdV (n = 9) infections, reaching objective response rates 74%, 79%, and 67%, respectively.^83^ The same group later reported the generation of a pentavalent product additionally targeting BKV and HHV6. The cells were administered to 38 HCT recipients with drug refractory CMV (n = 17), EBV (n = 2), AdV (n = 7), BKV (n = 16), and HHV6 (n = 3) infections. While VST products and patients matched at 1–7 HLA alleles, there were only 3 cases of grade-I, de novo GvHD. Ninety-two percent of patients responded to therapy, including patients with viral diseases; EBV-PTLD (n = 1), CMV colitis (n = 2), encephalitis (n = 1), AdV enteritis (n = 11), respiratory tract infection (n = 2), HHV6 encephalitis (n = 1), BKV-hemorrhagic cystitis (n = 14), and nephritis (n = 2). VST responses were confirmed to be of third-party donor origin in 11 cases and shown to persist in vivo for up to 12 weeks postinfusion.^87^

To facilitate the establishment of third-party cell banks and make pSTs broadly available, investigators sought to use donors with HLA types found at high frequency in the respective population (Figure 1). Given the multiple doses generated per each donor cell production, such banks established from as little as 25–30 carefully selected donors, could supply VSTs to all patients in need for urgent use based on HLA matching and provided 80% and 93% overall responses in refractory infections.^69,154^

More recently, the Baylor group generated a bank of CMV-STs from just 8 healthy donors. Donors were carefully selected based on an algorithm that compared 666 HCT recipient HLA types with the HLA types of a pool of diverse, healthy donors. This minibank was sufficient to provide VST matching at ≥2 HLA antigens to 28 of 29 patients referred.^66^ In addition, using a scale-up manufacturing protocol, by starting with a unit of blood it was feasible to generate >2000 VST doses for infusion.

As adoptive therapy with either third-party VSTs or transplant donor VSTs seems to trigger comparable antiviral responses that might be mediated by restoration of endogenous virus-specific immunity,^155^ off-the-shelf cell products are attracting increased interest. Currently, ElevateBio and Allovir biotech companies have joined forces to explore the use of off-the-shelf multi-VSTs as prophylaxis or treatment of CMV, AdV, EBV, BKV or HHV6 viral infections post-HCT (phase-2 or -3 trials, NCT04693637, NCT05305040, NCT05179057), as treatment of BKV infection post kidney transplant (phase-2, NCT04605484), as treatment of BKV-associated hemorrhagic cystitis post-HCT (phase-3, NCT04390113), or as treatment of respiratory viral infections from community acquired respiratory viruses (RSV, influenza, hMPV, and/or PIV) post-HCT (phase I/II NCT04933968).

An attractive alternative cell source for adoptive immunotherapy, especially as third-party, off-the-shelf approach, are the unconventional γδ T cells, an early innate arm of the immune system and key players in the fight against viruses. Their unique property of major histocompatibility complex (MHC)-independent recognition of their targets allows effective antitumor and antiviral control without GvHD. Their potential has mainly been explored within the context of cancer treatment, using either unmanipulated or genetically engineered γδ T cells by the introduction of αβ TCRs, chimeric antigen receptors (CARs), and drug-resistance genes.^156,157^ Gamma-delta T cells represent also an interesting treatment option for viral reactivations, yet overall understudied as a platform for antiviral immunotherapy.

### Pathogen-naive donors

pSTs can only be expanded from donors who have previously been exposed to pathogens thus having already shaped their immunological memory; given this, pST generation from antigen-seronegative donors, often CBU or younger donors, becomes highly challenging. Beyond the aforementioned third-party pST banks, several other strategies have also been employed to tackle this problem. First, by ex vivo priming of T cells, where T cells from pathogen-naive sources are ex vivo “educated” to recognize the targeted antigen(s) by priming and repeated stimulations with APCs in the presence of various cytokines.^139,142,158–161^ First results of the safety and efficacy profile of CBU-derived tri-VSTs look promising.^52^ A second approach is the donor vaccination. The rationale lies in the establishment of specific immunity by vaccination of a seronegative donor and subsequent generation and adoptive transfer of pSTs to the transplant recipient in cases of refractory and persistent viremia posttransplant.^162^ CMV-pp65 specific CTLs ex vivo expanded from a vaccinated CMV-sero-negative stem cell donor (Towne strain CMV vaccine) were administered in a haploidentical HCT recipient with a refractory CMV disease; CMV DNA decreased after the CTL infusion while CMV-specific cytotoxicity increased.^162^ However, this strategy has the limitations of usually not timely identification before transplantation of the most appropriate donor to be vaccinated early enough to develop immunological memory, the lack of effective vaccines against the commonest pathogens posttransplant and the logistical, ethical, and medical concerns on donor vaccination for the benefit of the recipient.^163^ A third approach to overcome the “pathogen-naive” donor issue is to generate genetically engineered pSTs with a transgenic TCR to redirect patient’s (autologous) or seronegative donor’s T cells against a desirable pathogen^164–175^ (Figure 1; Table 3).

**Table 3:** Genetically Modified pSTs

	Advanced Therapy Medicinal Product	Method	Reference
TCR-redirected	TCR-transgenic single-targeting VSTs (CMV-STs)	Vector-mediated knock in of a transgenic CMV TCR	Shub et al^169^
	TCR-transgenic single-targeting VSTs (CMV-STs)	Vector-mediated knock in of a transgenic CMV TCR	Wang et al^168^
	TCR-transgenic single-targeting VSTs (HBV-STs)	Vector-mediated knock in of a transgenic HBV TCR	Gehring et al^174^
	TCR-transgenic single-targeting VSTs (HBV-STs)	Knock in of a transgenic HBV TCR by electroporation	Meng et al^172^
	TCR-transgenic single-targeting VSTs (HBV-STs)	Knock in of a transgenic HBV TCR	Qasim et al^170^
	TCR-transgenic single-targeting VSTs (HCV-STs)	Vector-mediated knock in of a transgenic HCV TCR	Zhang et al^164^
	TCR-transgenic single-targeting VSTs (HIV-STs)	Vector-mediated knock in of a transgenic HIV TCR	Varela-Rohena et al^167^
	TCR-transgenic single-targeting VSTs (HIV-STs)	Vector-mediated knock in of a transgenic HIV TCR	Joseph et al^166^
	TCR-transgenic single-targeting VSTs (HPV-STs)	Vector-mediated knock in of a transgenic HPV TCR	Doran et al^175^
	TCR-transgenic single-targeting VSTs (HPV-STs)	Vector-mediated knock in of a transgenic HPV TCR	Jin et al^173^
	TCR-transgenic single-targeting VSTs (HPV-STs)	Vector-mediated knock in of a transgenic HPV TCR	Nagarsheth et al^171^
	TCR-transgenic single-targeting VSTs (EBV-STs)	Vector-mediated knock in of a transgenic EBV TCR	Zhang et al^176^
Drug-resistant	Tacrolimus-resistant single-targeting VSTs (EBV-STs)	FKBP12 knock down using small interfering RNA (siRNA)	De Angelis et al^177^
	Calcineurin inhibitors-resistant single-targeting VSTs (EBV-STs)	Retroviral transduction with a calcineurin mutant	Brewin et al^178^
	Calcineurin inhibitors-resistant single-targeting VSTs (EBV-STs)	IFN-γ secreting EBV-CTLs and retroviral transduction with a calcineurin mutant	Richiardeli et al^179^
	Glucocorticoid-resistant single-targeting VSTs (CMV-STs)	TALEN-mediated glucocorticoid receptor inactivation	Menger et al^180^
	Glucocorticoid-resistant single-targeting VSTs (CMV-STs)	CRISPR-mediated glucocorticoid receptor inactivation	Kaeufferle et al^181^
	Glucocorticoid-resistant multitargeting VSTs (CMV-, BKV-, AdV-STs)	CRISPR-mediated glucocorticoid receptor inactivation	Basar et al^182^
	Glucocorticoid-resistant multitargeting pSTs (CMV-, EBV-, AdV-, BKV-, AF-STs)	CRISPR-mediated glucocorticoid receptor inactivation	Koukoulias et al^183^
	Tacrolimus-resistant single-targeting VSTs (CMV-STs)	CRISPR-mediated FKBP12 knock out	Amini et al^184^
	Tacrolimus and MMF- transiently resistant TCR-transgenic single-targeting VSTs (HBV-STs or EBV-STs)	Electroporation of HBV-TCR or EBV-TCR mRNA and siRNA to knockdown FKPB1A	Hafezi et al^185^
	Glucocorticoid-resistant single-targeting VSTs (CoV-2-STs)	CRISPR-mediated GR inactivation	Basar et al^186^
	Tacrolimus-resistant single-targeting VSTs (CoV-2-STs)	CRISPR-mediated FKBP12 knock	Peter et al^187^
CAR-Ts specific for pathogens	AF-CAR-Ts	T cells redirected through CAR toward carbohydrate	Kumaresan et al^188^
	CMV-CAR-Ts	T cells redirected through CAR toward CMV glycoprotein B	Full et al^189^
	CMV-CAR-Ts	T cells redirected through CAR toward several proteins of CMV	Ali et al^190^
	EBV-CAR-Ts	T cells redirected through CAR toward EBV LMP1	Tang et al^191^
	HBV-CAR-Ts	T cells redirected through CAR toward HBV S or L protein	Bohne et al^192^
	HBV-CAR-Ts	T cells redirected through CAR toward HBV S protein	Krebs et al^193^
	HCV-CAR-Ts	T cells redirected through CAR toward HCV E2 glycoprotein	Sautto et al^194^
	HIV-CAR-Ts	HIV-specific T cells	Walker et al^195^
	HIV-CAR-Ts	T cells redirected through CAR toward HIV env	Mitsuyasu et al^196^
	HIV-CAR-Ts	T cells redirected through CAR toward HIVgp120	Deeks et al^197^
	HIV-CAR-Ts	HIV-resistant T cells redirected through CAR toward HIV	Hale et al^198^
	HIV-CAR-Ts	Broadly neutralizing antibody-derived (bNAb-derived) CAR T-cell therapy	Liu et al^199^
	HIV-CAR-Ts	Multi-specific anti-HIV duoCARs	Anthony-Gonda et al^200^
	HIV-CAR-Ts	Re-engineered CD4-based	Leibman et al^201^
pSTs coupled with a CAR	EBV-STs armed with CAR-Ts	Proof of principle for ex vivo expanded EBV-STS armed with a 1st generation GD2 (disialoganglioside)-CAR	Rossig et al^202^
	EBV-STs armed with CAR-Ts	Ex vivo expanded EBV-STS armed with a 1st generation CD30-CAR	Savoldo et al^203^
	Patient-derived tri-VSTs armed with CAR-Ts	Ex vivo expanded tri-VSTs (EBV-, CMV-, AdV-STs) armed with a 1st generation GD2 (disialoganglioside)-CAR	Pule et al^204^
	HCT donor-derived tri-VSTs armed with CAR-Ts	Ex vivo expanded tri-VSTs (EBV-, CMV-, AdV-STs) armed with a 2nd generation CD19-CAR	Cruz et al^205^
	tri-VSTs armed with CAR-Ts	Ex vivo expanded tri-VSTs (EBV-, CMV-, AdV-STs) armed with a 1st generation GD2 (disialoganglioside)-CAR	Sun et al^206^
	CMV-STs armed with CAR-Ts	IFN-γ capture-enriched CMV-STs armed with a 2nd generation CD19-CAR and combined with a CMV vaccine	Wang et al^207^
	HCT donor-EBV-STs armed with CAR-Ts	Ex vivo expanded EBV-STS armed with a 1st generation CD30-CAR	Rossig et al^208^
	HCT donor-derived tri-VSTs armed with CAR-Ts	Ex vivo expanded tri-VSTs (EBV-, CMV-, AdV-STs) armed with a 2nd generation CD19-CAR	Lapteva et al^209^
	CMV-CD19 CAR-Ts	IFN-γ capture enriched CMV-STs armed with a 2nd generation CD19-CAR	Wang et al^210^

AdV = adenovirus; AF = Aspergillus fumigatus; BKV = BK virus; CAR-Ts = chimeric antigen receptor T cells; CMV = cytomegalovirus; EBV = Epstein-Barr virus; HHV6 = human herpesvirus 6; HIV = human immunodeficiency virus; hMPV = human metapneumovirus; HPV = human papillomavirus; HPIV3 = human parainfluenza virus-3; HSV-1 = herpes simplex virus type 1; IFN-*γ* = interferon-*γ*; MCPyV = Merkel cell polyomavirus; PIV = parainfluenza virus; RSV = respiratory syncytial virus; SARS-CoV-2 = severe acute respiratory syndrome coronavirus 2; VZV = varicella zoster.

However, this approach entails several challenges, including (i) limited access to patients expressing specific HLAs; (ii) epitope restriction potentially increasing the risk of immune escape; (iii) risk for TCR mispairing between the endogenous and the transgenic TCR, mounting a potential harmful specificity^211,212^; and (iv) the absence of normal negative regulation, such as TCR downregulation in proportion to the strength of initial antigen recognition, stemming from the extrinsic promoter-driven expression of transgenic TCR.^213^ Orthotopic TCR replacement by a transgenic one may resolve some of the above issues and enable TCR-based therapies.^214^

### HLA restriction

The HLA restriction is another brake to broader pSTs applicability, limiting the administration of pSTs to HLA-matched recipients. To overcome this hurdle, the MHC-independent chimeric antigen receptor T cells (CAR-Ts) have also been proposed against infections and infectious diseases. To date, few anti-infectious CARs against CMV,^189,190^ EBV,^191^ hepatitis B virus (HBV),^25,192^ HCV,^194^ and AF^188^ have been described (Figure 1; Table 3). These proof-of-principle studies suggest that the application of CAR-Ts might extend beyond cancer to both viral and fungal infections or even couple the antipathogen with the antitumor response by generating bi-specific T cells recognizing both a pathogen and a tumor marker (as discussed later below)^202,203,210,215–217^; yet further studies are required to overcome technical challenges, identify optimal surface proteins as targets, and improve safety and efficacy.

### Sensitivity of pSTs to immunosuppressive drugs

In patients receiving high-dose and long-term immunosuppression, such as HCT patients with GvHD or SOT patients, who are in fact, the most vulnerable to opportunistic infections, pSTs become functionally impaired in vivo.^218–222^ This creates the paradox of precluding from the potential benefits of adoptive immunotherapy, the patients who are most likely to experience severe infections and are those with an unmet need for effective treatment of opportunistic infections.

To this end, several groups have been exploring mechanisms to genetically engineer pSTs and render them resistant to immunosuppressants (Table 3). Two teams have simultaneously introduced the generation of EBV-STs with resistance to calcineurin inhibitors (tacrolimus, FK506, or/and cyclosporin A), the most widely used immunosuppressive agents in the transplant setting, using either a specific small interference RNA (siRNA) to knock down the FK506-binding protein (FKBP12)^177^ or calcineurin mutants to disrupt its docking to calcineurin inhibitors.^178,179^ The latter approach is being currently evaluated in an open-label, nonrandomized, multicenter phase I trial assessing the safety of tacrolimus-resistant autologous EBV-STs in SOT patients with PTLD (NCT03131934).

The recent emergence of highly versatile genome-editing technologies has provided additional tools for rapid and affordable manufacturing of gene manipulated pSTs. In addition to FKBP12, the glucocorticoid receptor has also been targeted for inactivation by both transcription activator–like effector nucleases (TALENs) and clustered regularly interspaced short palindromic repeats (CRISPR)-CRISPR-associated protein 9 (Cas9). We and others have generated either glucocorticoid-resistant single-targeting VSTs,^180,181^ multitargeting VSTs,^182^ multi-pSTs,^183^ or tacrolimus-resistant single-targeting VSTs^184^ (Table 3), being able to expand and actively function even in the presence of intense immunosuppression posttransplant. Clinical studies are underway to assess their safety and preliminary efficacy (NCT05101213).

### Antigenic competition

Antigenic competition represents the major challenge for the generation of a single T-cell product targeting a broad spectrum of pathogens that arises from shared antigen-bearing APCs, as well as from the varying (high or low) frequencies of subsets of antigen-specific T cells in the starting PBMC population.^223,224^ Antigenic competition may favor the preferential outgrowth of high frequency VSTs (such as those directed against EBV and CMV antigens) over the low frequency antigen-specific T cells (such as those directed against AF and BKV), resulting in products dominated by responses to a single or a restricted repertoire of pathogens. Although in several reports, antigenic competition noticeably decreased the frequency of antigen-specific T cells in a multi-pST product as compared to single-targeting pSTs,^50,144,146,225^ other reports have shown that antigenic competition could be mitigated by the direct stimulation of PBMCs with pepmixes and culture conditions.^13,53,57^ Indeed, multivalent cell products simultaneously targeting a broad spectrum of pathogens (up to 7)^58^ could expand in vivo, in response to viral challenge and produce clinically beneficial effects against multiple pathogens (Table 1). However, the number of antigens to be targeted by a single-cell product could not be unlimited and should be determined on the basis of the clinical need, balancing between a broad, as possible, coverage against pathogens, manufacturing costs, and possible antigenic competition.

### pSTs as prophylaxis or treatment?

Recently, letermovir as primary prophylaxis to prevent clinically significant CMV infection in HCT seropositive patients has been associated with significant clinical benefit, leading to decreased clinically significant CMV reactivation both in a phase III trial^226^ as well as in the real world.^227^ Therefore, prophylactic administration of pSTs for CMV reactivation prevention may not be necessary. However, mutations in the genes encoding for the CMV terminase complex may confer resistance of the virus to letermovir, and in this context, VSTs remain important in limiting drug-refractory CMV disease. Moreover, letermovir prophylaxis may be associated with impaired CMV-specific immunity.^228^ In patients who lack effective CMV-specific T-cell immunity, delayed-onset CMV infection has been shown to occur at higher frequency compared to patients with reactivation following letermovir^229^; thus, there may be room for pST therapy in those patients. As far as other dsDNA viruses are concerned (ie, AdV, EBV, HHV6, BKV, JC virus), there are limited if any, antiviral drugs available. Current medical prophylaxis regimens are ineffective at preventing viral reactivation and often associated with toxicities. Therefore, the prophylactic administration of pSTs against these viruses in high-risk patients early post-HCT, remains rational. However, given as treatment, pSTs may be more cost-effective as some patients may not develop infections or acute GvHD requiring high-dose steroids may occur before infection, thus abrogating the pSTs potential.

## EXTENDING THE USE OF PSTS BEYOND THE TRANSPLANT SETTING

The compelling results on the management of viral complications posttransplant with adoptive immunotherapy has stimulated research with the use of pSTs in other immunocompromised patient populations, outside the context of transplantation.

### Less-immunogenic EBV-associated diseases

The impressive results of EBV-STs in type II latency tumors posttransplant, has attracted researchers’ interest to the evaluation of adoptive transfer of EBV-STs as treatment of less-immunogenic EBV-related malignancies, occurring outside the transplantation context. Despite the low immunogenicity of the targeted antigens, autologous EBV-STs, either nonengineered^230–232^ or genetically modified with a dominant-negative TGF-β receptor to become resistant to otherwise inhibitory concentrations of TGF-β in the tumor microenvironment,^233^ could be generated from patients with type 2 latency tumors. Upon administration, EBV-STs elicited objective clinical benefit in patients with Hodgkin- and non-Hodgkin lymphomas^230–233^ or nasopharyngeal carcinoma (NPC)^234–237^ without major toxicities.

EBV-STs have also been proposed as an alternative cell-based approach for the management of multiple sclerosis (MS). Indeed, EBV has been very recently proposed as the leading cause of MS.^238^ Preliminary results from a phase I study have reported sustained clinical improvement in 7 of 10 patients with MS treated with autologous EBV-STs, without experiencing any serious adverse event, including disease exacerbation.^239,240^ Therefore, an academic phase-I (Nantes University Hospital) and a commercial phase-I/II (Atara Biotherapeutics: product ATA188) clinical trial, assessing the safety and feasibility or/and efficacy of adoptive cell therapy with autologous or allogeneic EBV-STs in patients with a first clinical episode highly suggestive of MS or progressive MS, respectively, are currently running (NCT02912897 and NCT03283826).

### Primary immunodeficiency disorders

Patients with primary immunodeficiency disorders (PIDs), a group of congenital defects of immunity such as severe combined immunodeficiency (SCID), are susceptible to opportunistic infections frequently caused by common pathogens such as CMV, EBV, AdV, VZV, and respiratory viruses, even before HCT.^241,242^ Theoretically, in this patient population, pSTs could serve as a bridging therapy before HCT. So far, only 6 of 63 patients with PID treated with pST therapy, received the cell products before HCT.^243^ Notwithstanding the very small number of patients, this study provided the first evidence that individuals with PID could benefit, albeit at higher doses, from adoptive immunotherapy with pSTs, and proceed to transplant expeditiously.

### HPV-associated cancer

In a similar context, Hinrichs’s group developed autologous genetically engineered TCR-Ts directed against the HPV-E6^175^, and recently -E7 antigen,^171,173^, demonstrating robust tumor regression and objective clinical responses in 6 of 12 patients, including 4 of 8 patients with anti-PD-1 refractory disease in a first-in-human phase I trial in collaboration with Kite-Gilead. E7 TCR-T cells are currently being tested in the ph-II arm of that trial (NCT02858310) in patients with metastatic HPV-associated epithelial cancers. In an academic setting, the Baylor group is clinically investigating the safety and efficacy of patient-derived HPV-STs, which are being ex vivo expanded to target both the E6- and E7- antigens through their native TCR, in patients with relapsed HPV-associated cancers. HPV-STs are also genetically modified to become TGF-beta resistant and combined with the immune-checkpoint inhibitor Nivolumab, to reduce antigen escape-mediated relapses and T-cell exhaustion (NCT02379520).

### Merkel cell carcinoma

There are limited data on adoptive transfer of VSTs in Merkel cell carcinoma (MCC), an aggressive skin cancer, typically caused by the Merkel cell polyomavirus (MCPyV). Chapuis’s group first observed a complete response in 2 of 3 metastatic lesions of a 67-year-old man with metastatic MCPyV-expressing MCC who was treated with autologous ex vivo expanded, HLA-A*2402-epitope-restricted specific T cells (MCPyV-STs).^88^ They later treated 2 more patients with autologous HLA B*3502- and HLA-A*0201-epitope-restricted MCPyV-STs.^89^ Notwithstanding the observed tumor lesion regressions, both patients relapsed, thus confirming the risk of tumor escape from single-epitope-targeting T cells. In the gene-engineered T-cell field, Bluebird bio and Fred Hutchinson Cancer Research Center are actively recruiting patients with metastatic or unresectable MCPyV-associated MCC to be treated with autologous A2-MCC1-expressing TCR-Ts, specific for an HLA-A02-restricted viral oncoprotein (NCT03747484).

### HBV-related hepatocellular carcinoma

The proof-of-concept of using TCR-Ts redirected to target HBV antigen was assessed in a patient who developed hepatocellular carcinoma (HCC) relapse 10 years following liver transplantation for HBV^+^ HCC.^170^ A single infusion of only 10^4^ per kg HBsAg-specific TCR-Ts led to a reduction of serum HBsAg levels by >90% within 30 days, without any detectable on/off-target toxicities. Importantly, the risk of inducing a severe liver injury due to on-target, off-tumor lysis of normal infected hepatocytes was minimized in this patient in whom HBsAg was exclusively produced by HCC extrahepatic metastasis and not by the transplanted liver; yet a possibility that it should always be taken into consideration. Lion TCR biotechnology company reported the first results of this approach on 8 patients who had not met the criteria for liver transplantation, showing a reduction or stabilization of circulating HBsAg and HBV DNA levels in most of the patients after HBV-TCR-T-cell infusion,^172^ while post-liver transplantation data are still pending (NCT02719782). Importantly, immunosuppressive drug-resistant HBV-TCR-Ts have been recently developed for immune therapy of HCC in liver transplant patients.^185^

### Progressive multifocal leukoencephalopathy

Progressive multifocal leukoencephalopathy (PML) is a devastating and generally fatal neurologic disease occurring in profoundly immunocompromised patients due to brain infection with JC polyomavirus and lacking effective treatment, unless cellular immunity is restored.^244^ As BKV and JC virus are closely related and share several immunogenic proteins, adoptive transfer of allogeneic JC-STs or BKV-STs has also been explored as treatment of PML.^49,65,76–78,86^ Preliminary, albeit very promising, results of those trials demonstrated safety of the infusion of partially matched VSTs as well as alleviation of clinical symptoms, progressive decline of viral load in cerebrospinal fluid and an unprecedented survival beyond 1 year in a number of PML patients with poor expectation for survival.^49,65,76–78,86^

### Human immunodeficiency virus

Although antiretroviral therapy (ART) is successfully used to suppress HIV-1 replication, viral latency is responsible for the persistence of a reservoir of infected CD4+ T cells which lead to systemic viremia should ART is ceased, thus constituting major barrier to HIV cure.^245^ Functional HIV-specific T cells (HIV-STs), both natural^91–96,128,129,246^ or genetically redirected,^166,167,194–200^ have been manufactured from HIV+ or HIV-seronegative donors (Table 3) and are currently being evaluated in early-stage immunotherapy trials as a strategy to eradicate the HIV reservoir and prevent HIV rebound.

### Coronavirus disease

The urgency of the pandemic has prompted scientists from different fields (pharmaceuticals, vaccines, cellular therapies) to address the problem. Leveraging the VST platform has been recently proposed *as* treatment against the severe acute respiratory syndrome coronavirus 2 (SARS-CoV-2) and its associated life-threatening coronavirus disease (COVID-19)^80,132–138^ (Table 2). In the clinical setting, 7 groups including ours, have been evaluating off-the-shelf SARS-CoV-2-specific T cells (CoV-2-STs) derived from convalescent donors as treatment for high-risk COVID-19 patients (EudraCT:2021-001022-22/NCT05447013, NCT04351659/NCT04457726, NCT04578210, NCT04762186, NCT04896606, NCT04742595, NCT04765449), whereas 1 group uses stem cell donor-derived CoV-2-STs as prophylaxis against SARS-CoV-2 in individuals receiving allo-HCT (NCT05141058). Thus far, the Madrid team has published the phase I results showing that infusion of memory T cells from convalescent donors was safe at all tested doses and resulted in lymphocyte recovery 2 weeks postinfusion in 9 treated patients.^247^ Repeated off-the-shelf CoV-2-ST infusions (Allovir’s product ALVR109) also resulted in clinical and virologic improvement when administered to treat refractory severe COVID-19 pneumonia in a heart transplant patient.^81^ Our group assessed in a randomized 2:1 phase I/II trial, the safety and efficacy of the administration of ex vivo expanded, partially HLA-matched CoV-2-STs in addition to standard of care (SoC) in 57 patients with severe COVID-19 as compared to 30 patients who received SoC-only (control arm) (NCT05447013). The add-on treatment with CoV-2-STs was well tolerated and increased the likelihood of recovery by day 30, shortened the time to recovery and lowered by 51% the risk of mortality than SoC-alone, suggesting that the off-the-shelf immunotherapy with CoV-2-STs can serve as a safe and effective treatment in a real-world environment for severe COVID-19.^79^

To optimize CoV-2-ST performance in the presence of immunosuppressants which are used for the management of severe COVID-19, genetically modified CoV-2-STs resistant to glucocorticoids^186^ or tacrolimus^187^ have also been proposed (Table 1).

## UNLEASHING THE POTENTIAL OF PSTS

CAR-Ts have achieved unprecedented success in patients with previously incurable relapsed/refractory B-cell malignancies. Nonetheless, their poor persistence attributed to the frail fitness of patient-derived T cells, the phenotype (proportion of CD4+ versus CD8+ T cells, less memory-like cell phenotype), and the exhaustion due to sustained antigenic stimulation in vivo, represents the main reason for antigen-positive relapse and a major limitation.^248–251^

Unlike CAR-Ts, pSTs have been shown to persist for up to 9 years.^9^ The remarkable difference in their lifespan mainly stems from the fact that pSTs are nonengineered cells “per se” and as “natural” pSTs, their transmitting signaling via native TCR involved in cognate antigen recognition and T-cell activation, remains unadulterated. In contrast, artificial CARs result in an abnormal or rewired TCR signaling^252,253^ as well as an unconstrained ligand-independent constitutive signaling, known as tonic signaling,^254^ being incriminated in the exhaustion, poor persistence or the toxicities associated with CAR-Ts^255^. Indeed, CD19-CAR-T cells with disrupted endogenous TCR, although similarly functional to TCR+/CAR-Ts, presented only limited persistence in vivo.^256^ Another important factor on the difference in persistence between the engineered and nonengineered, antigen-specific T-cell products, is the starting source of T cells; healthy donor- and quiescent memory pool-derived pSTs have a clear intrinsic survival advantage over CAR-Ts, which mainly derive from lymphopenic, usually heavily pretreated patients and contain higher frequencies of effector memory cells. Numerous studies have shown that T-cell products enriched for early lineage cells expand better and may delay CAR T-cell exhaustion, leading to improved persistence of the CAR-Ts.^251^

To overcome the significant hindrance of limited CAR-T function and persistence, the Baylor group proposed the introduction of CARs (CD30-, GD2- or CD19-CAR) into EBV- or tri-VSTs (either patient- or HCT donor-derived), as a platform to generate CAR-equipped pSTs^202–205,209,257^ (Table 3) presenting the ability to expand in the presence of viral antigens through their native TCR, while producing some objective clinical responses through CAR^204,209,258^ or/and after boosting the antitumor activity with vaccination.^207,210^ However, the latter was not confirmed in a clinical trial with 11 pediatric patients with relapsed acute lymphoblastic leukemia who received EBV-STs bearing CD19-CAR and were boosted with vaccination; despite the enhanced persistence of adoptively transferred T cells in vivo, their expansion was poor and antileukemic activity limited.^208^ Nonetheless, it has become clear that the transfer of pSTs coupled with a CAR is safe, without inducing GvHD, cytokine release syndrome or neurotoxicity, thus confirming the more “physiological” function of nonengineered pSTs. Importantly, the minimized risk for alloreactivity, associated with native TCR engagement to latent virus antigens, makes the dual, specific TCR- and CAR- T cells ideal products for off-the-shelf therapy with CAR-Ts. To date, ongoing clinical trials assess the safety and efficacy of autologous dual TCR- and CAR-specific T cells with (NCT01192464, NCT00709033) or without vaccination boosting (NCT05432635) or allogeneic dual TCR/CAR-specific T cells (NCT01430390, NCT04288726). In parallel, Atara Bio expects IND for off-the-shelf, allogeneic CD19-CAR EBV-STs (product A3219) in the last quarter of 2022. If proved safe and effective, this platform will improve longevity of remissions after CAR-T-cell therapy in the future.

## CONCLUDING REMARKS

The ability of adoptive immunotherapy to restore antiviral immunity and clear viral infections as a “living drug” platform, has been unequivocally shown and over the last three decades, the manufacture and clinical application of antigen-specific T cells has greatly evolved. The high and durable responses of pathogen-specific T cells, the broad coverage of antiviral spectrum, the good safety profile, the fast manufacturing, the increasing applicability due to third-party cell banks, have introduced a new era in the management of opportunistic infections. pSTs, eventually become part of the conventional antiviral treatment, falling under ECIL’s recommendations as regards EBV-PTLD and refractory CMV infection or disease post-HCT. Nevertheless, for cellular therapies, the strength of recommendation in combination with the level of evidence does not exceed level BIIu in refractory CMV disease^259^ or BIII as the second-line therapy for EBV-PTLD,^260^ implicating that such approaches, albeit a major breakthrough, have not yet become a standard treatment. This could be attributed to the fact that the majority of clinical trials conducted so far were early phase clinical trials, under academic grant funding, including in their majority limited number of patients. However, the progress made the last decade on simplifying the manufacturing process and speeding up the production along with the extended applications beyond the transplant setting, have attracted the interest of biotech and pharmaceutical companies and paved the way for late phase, controlled, prospective clinical trials studying the efficacy of adoptive immunotherapy. The results from Trace (“TRansfer of Adenovirus, Cytomegalovirus and Epstein-Barr virus-specific T cells”—NCT04832607), the first, multinational, ph-III trial, sponsored by the European Commission, and other nonrandomized (NCT03394365) or randomized ph-III trials sponsored by Atara Biotherapeutics, Allovir (NCT05305040, NCT04390113, NCT05179057) and Tessa Therapeutics (NCT02578641) are expected to generate strong clinical evidence on safety and efficacy of adoptive immunotherapy and enable the industrial investment and ultimately, regulatory approval. To facilitate academic medicine developers targeting an unmet medical need to optimize their development plans, European Medicines Agency (EMA) has recently launched the “PRIME” project, offering early and proactive support at improving clinical trial designs and enabling accelerated assessment of medicines application (https://www.ema.europa.eu/en/human-regulatory/research-development/prime-priority-medicines).

By the future incorporation of adoptive transfer of pSTs into clinical practice and acquisition of real world data, the adoptive transfer of pSTs may obtain a higher level of recommendation and evidence in the treatment’s guidelines. Moreover, patient risk stratification by monitoring the pathogen-specific immune reconstitution posttransplant by functional assays can guide the clinical management of patients at high-risk for morbidity and mortality from opportunistic reactivations and select the best candidates for pathogen-specific immunotherapy.^261^ Adoptive immunotherapy with pSTs may ultimately become a standard treatment for opportunistic infections post-HCT while serve as a platform for targeting novel emerging pathogens in the future.

## AUTHOR CONTRIBUTIONS

Conceptualization, writing, review and editing, AP, MA, GK, IT and EY.

## DISCLOSURES

The authors have no conflicts of interest to disclose.

## SOURCES OF FUNDING

Funding for this project was provided by the T2EDK-02437 Research, Technology Development and Innovation (RTDI) State Aid Action “RESEARCH - CREATE -INNOVATE.”
